# Intertransverse Process Block With Catheter Placement for Postoperative Pain Management in a Patient With Alcoholic Liver Disease and Portal Hypertension: A Case Report

**DOI:** 10.7759/cureus.80788

**Published:** 2025-03-18

**Authors:** Keisuke Nakazawa, Ayano Takenaka, Takahiro Suzuki

**Affiliations:** 1 Department of Anesthesiology, Nihon University School of Medicine, Tokyo, JPN

**Keywords:** alcoholic liver cirrhosis, catheter technique, epidural venous plexuses, intertransverse process block, intertransverse tissue complex, paravertebral by proxy, portal hypertension, postoperative analgesia, regional anesthesia

## Abstract

Epidural analgesia is typically avoided in patients with portal hypertension due to multiple risk factors: engorgement of epidural venous plexuses, platelet dysfunction despite normal counts, and potential postoperative coagulopathy following liver surgery. These risks persist even when preoperative coagulation parameters appear normal. While peripheral nerve blocks are increasingly utilized for minimally invasive laparoscopic procedures, intertransverse process block (ITPB) with catheter placement offers a high-quality analgesic strategy that supports early ambulation and postoperative recovery with a significantly reduced risk profile in such patients.

A 76-year-old male patient with alcoholic liver cirrhosis (Child-Pugh class A) and a history of esophageal variceal bleeding underwent laparoscopic partial hepatectomy of segment 3 for suspected hepatocellular carcinoma. Despite normal coagulation parameters (prothrombin time-international normalized ratio 1.1 and activated partial thromboplastin time 33 seconds), epidural analgesia was contraindicated due to portal hypertension with multiple vascular anomalies. Bilateral ultrasound-guided ITPB was performed at the Th8-9 level with catheter placement in the intertransverse tissue complex. Analgesia was maintained with intermittent boluses of 0.25% levobupivacaine (10 mL bilaterally, twice daily) for three postoperative days, supplemented with intravenous patient-controlled analgesia (IV-PCA) fentanyl (baseline infusion 10 μg/hour, bolus dose 10 μg, lockout time 10 minutes). The patient reported minimal pain scores (numerical rating scale 0-2 at rest, 2-3 with movement), achieved early mobilization, and did not require any PCA boluses throughout recovery. Cold testing confirmed adequate sensory blockade from Th8 to Th11 on each postoperative day until catheter removal.

ITPB with catheter placement provided safe and effective analgesia in a patient with portal hypertension, enabling early mobilization and rehabilitation without risking complications associated with epidural techniques. This approach represents a viable alternative to epidural analgesia in high-risk patients with compromised liver function and vascular abnormalities.

## Introduction

Epidural analgesia has been the gold standard for pain management in abdominal surgery due to its effectiveness in reducing postoperative pain and opioid consumption. However, in patients with portal hypertension, epidural procedures carry significant risks due to multiple factors: engorgement of the epidural venous plexus [1], platelet dysfunction despite normal counts, and potential postoperative coagulopathy following liver surgery. These patients often develop extensive portosystemic collaterals that increase the risk of epidural hematoma formation, even when preoperative coagulation parameters appear normal [2].

Recent advances in ultrasound-guided regional anesthesia have introduced safer alternatives to neuraxial blocks. The concept of "paravertebral by proxy" techniques has emerged, achieving similar analgesic effects without direct needle placement in the paravertebral space [3]. An intertransverse process block (ITPB) is one such technique that targets the intertransverse tissue complex (ITTC), which serves as a gateway to the paravertebral space [4].

ITPB involves the injection of a local anesthetic posterior to the superior costotransverse ligament between adjacent transverse processes. This provides analgesia by spreading it to the paravertebral space while avoiding the risks associated with direct paravertebral or epidural puncture. The technique maintains a safe distance from vascular structures and the pleura, making it particularly valuable in patients with coagulopathy or vascular abnormalities [5].

Furthermore, placing catheters in the ITTC allows for prolonged analgesia through intermittent bolus administration, which can facilitate early mobilization and rehabilitation in the postoperative period. This case report demonstrates the successful use of bilateral ITPB with catheter placement for postoperative pain management in a patient with alcoholic liver disease and portal hypertension undergoing laparoscopic partial hepatectomy.

## Case presentation

A 76-year-old male patient (height 167 cm, weight 72 kg) with a medical history of alcoholic liver cirrhosis (Child-Pugh class A) and previous esophageal variceal bleeding was scheduled for laparoscopic partial hepatectomy of segment 3 for suspected hepatocellular carcinoma (HCC). Preoperative pulmonary function testing revealed normal vital capacity (VC 3.73 L, %VC 115%) but moderate obstructive ventilatory impairment (forced expiratory volume in one second 62.5%), consistent with the patient's 60-year smoking history (20 cigarettes/day for 60 years). Preoperative laboratory data showed normal coagulation parameters (prothrombin time-international normalized ratio 1.1, activated partial thromboplastin time 33 seconds, platelet count 156 × 10³/µL), as detailed in Table 1.

**Table 1:** Preoperative laboratory results PT-INR: prothrombin time-international normalized ratio; PT: prothrombin time; AST: aspartate aminotransferase; ALT: alanine aminotransferase; eGFR: estimated glomerular filtration rate

Parameter	Patient's value	Reference range
WBC	5,200/μL	3,500-9,000/μL
RBC	4.1 × 10^6^/μL	4.0-5.5 × 10^6^/μL
Hematocrit	37%	40%-54%
Platelets	161 × 10^3^/μL	150-450 × 10^3^/μL
Total protein	6.6 g/dL	6-8 g/dL
Albumin	3.8 g/dL	3.5-5 g/dL
CRP	0.1 mg/dL	<0.3 mg/dL
Sodium	138 mmol/L	135-145 mmol/L
Potassium	3.8 mmol/L	3.5-5 mmol/L
Chloride	104 mmol/L	98-107 mmol/L
Creatinine	0.8 mg/dL	0.6-1.2 mg/dL
eGFR	66.9 mL/minute/1.73 m^2^	>90 mL/minute/1.73 m^2^
AST	26 U/L	10-40 U/L
ALT	15 U/L	5-45 U/L
Total bilirubin	0.5 mg/dL	0.2-1.0 mg/dL
PT activity	85%	70%-130%
PT-INR	1.1	0.9-1.1

**Figure 1:**
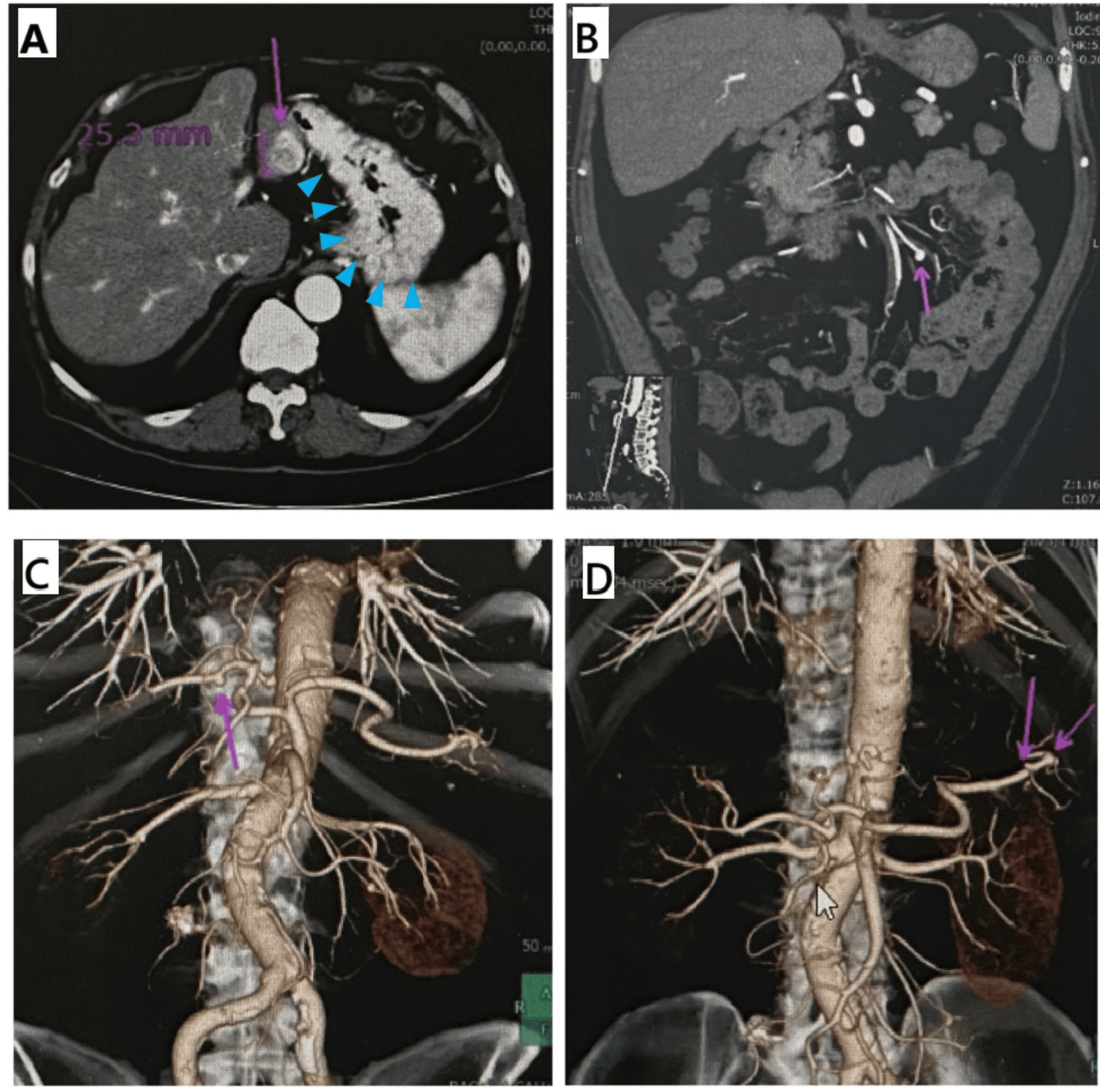
Preoperative CT images demonstrating HCC and vascular anomalies. (A) A 25-mm early enhancing lesion in liver segment 3 suggestive of HCC (purple arrow) and gastric varices (blue arrowheads). (B) Mesenteric arterial aneurysm (purple arrow). (C) Right hepatic arterial aneurysm (purple arrow). (D) Multiple aneurysms in the distal arterial trunk (purple arrows). These images demonstrate the extensive vascular abnormalities associated with the patient's portal hypertension HCC: hepatocellular carcinoma

Preoperative CT imaging revealed a 25-mm early enhancing lesion with washout and pseudocapsule in liver segment 3, consistent with HCC. The scan also showed significant portosystemic collaterals, including gastric varices and multiple arterial aneurysms in the mesenteric artery, right hepatic artery, and distal arterial trunk (Figure 1).

The patient had previously undergone clipping for duodenal varices and balloon-occluded retrograde transvenous obliteration. General anesthesia was induced and maintained using standard monitoring (electrocardiogram, noninvasive blood pressure, pulse oximetry, capnography, temperature, and bispectral index monitoring). A radial arterial line was placed for continuous blood pressure monitoring and blood sampling. Anesthesia was achieved with propofol (target-controlled infusion), fentanyl, remifentanil, and rocuronium for muscle relaxation.

The anesthesia team decided to avoid epidural analgesia due to suspected portal hypertension with extensive portosystemic collaterals. The surgery was performed under general anesthesia and lasted 312 minutes with minimal blood loss (50 mL).

**Figure 2:**
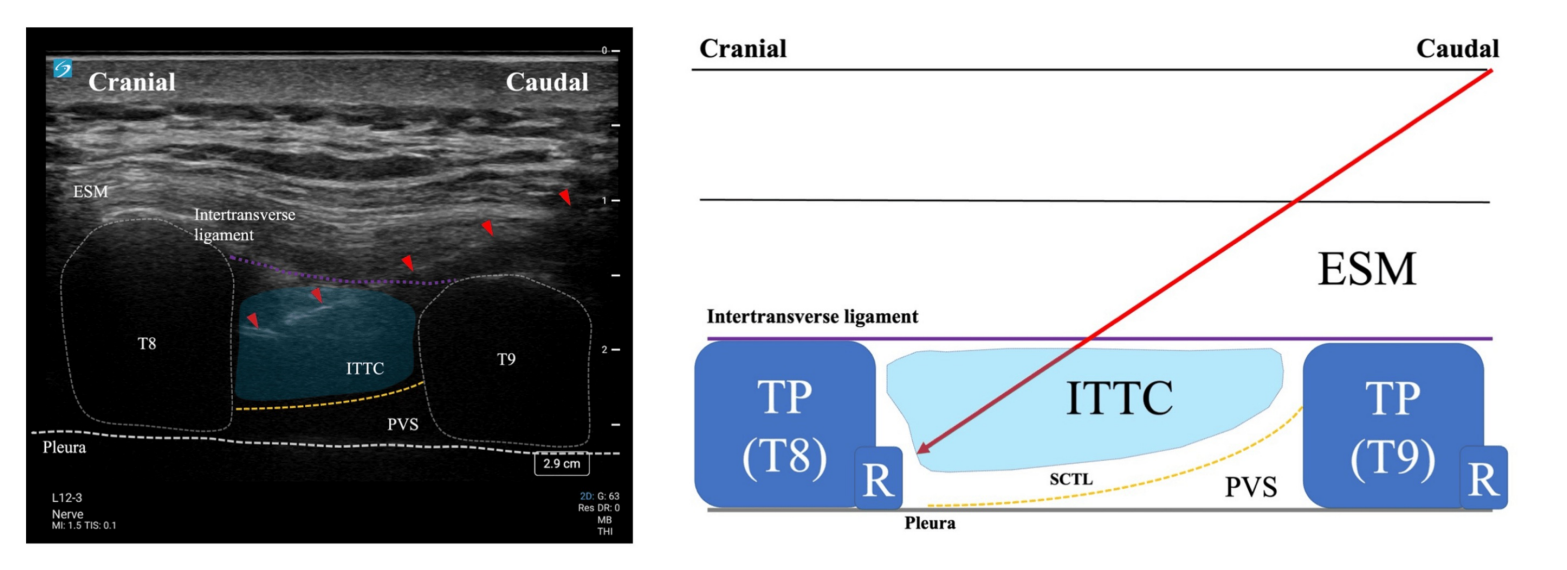
Ultrasound anatomy of the ITPB. Ultrasound image (left) and corresponding schematic (right) of the intertransverse process block at the T8-T9 level. The ultrasound image shows a parasagittal view with key anatomical structures: ESM, T8, and T9 transverse processes (outlined with white dotted lines), ITTC (colored in light blue), PVS, and pleural line. The purple dotted line indicates the intertransverse ligament, while the yellow dotted line represents the SCTL. Red arrowheads mark the needle trajectory approaching from caudal to cranial direction. The light blue area illustrates the spread of local anesthetic within the ITTC. The schematic on the right provides a simplified anatomical representation of the same structures to enhance understanding of the sonoanatomy and needle path (red arrow) for the ITPB technique ITPB: intertransverse process block; TP: transverse process; R: rib; ESM: erector spinae muscle; ITTC: intertransverse tissue complex; PVS: paravertebral space; SCTL: superior costotransverse ligament

Following emergence from anesthesia and confirmation of stable vital signs, bilateral ITPB was performed at the Th8-9 level under ultrasound guidance with the patient in the left lateral position. Using a high-frequency linear probe, the ITTC was identified (Figure 2).

**Figure 3:**
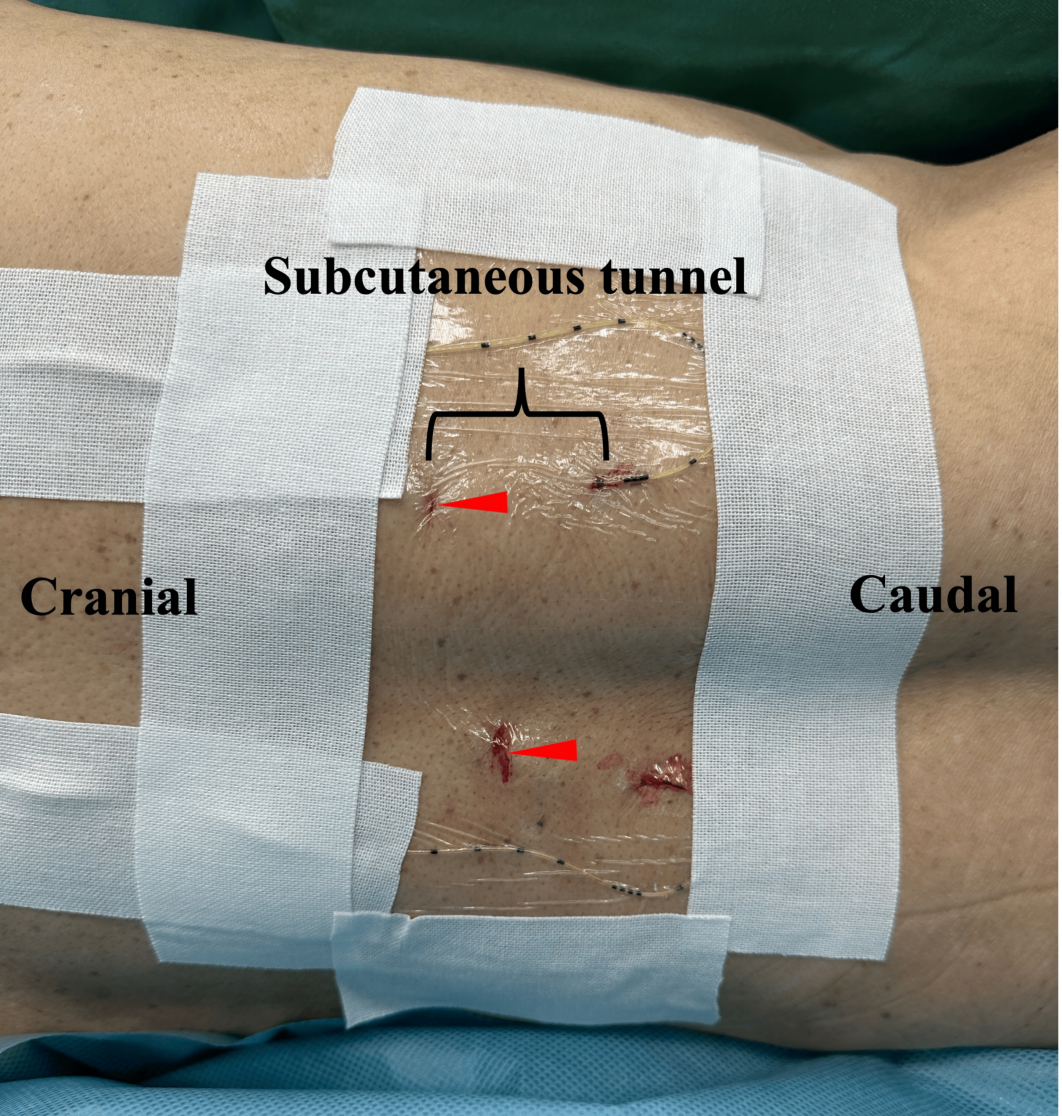
Bilateral ITPB catheter placement with subcutaneous tunneling Patient positioning and catheter placement. Postoperative photograph showing bilateral ITPB catheters tunneled subcutaneously. The ITPB catheters were inserted with the patient in the left lateral position. Red arrowheads indicate the needle insertion sites, with catheter tips placed within the ITTC. The subcutaneous tunnel was created approximately 5 cm caudally from the insertion sites for secure fixation ITPB: intertransverse process block; ITTC: intertransverse tissue complex

After sterile preparation, 18-gauge Tuohy needles were inserted using an in-plane technique. The needle tips were advanced intentionally into the ITTC, and epidural catheters were threaded 3-4 cm beyond the needle tip. The total distance from the skin to the ITTC was approximately 8 cm. The catheters were secured bilaterally and tunneled subcutaneously for 5 cm to reduce infection risk and prevent local anesthetic leakage (Figure 3).

**Figure 4:**
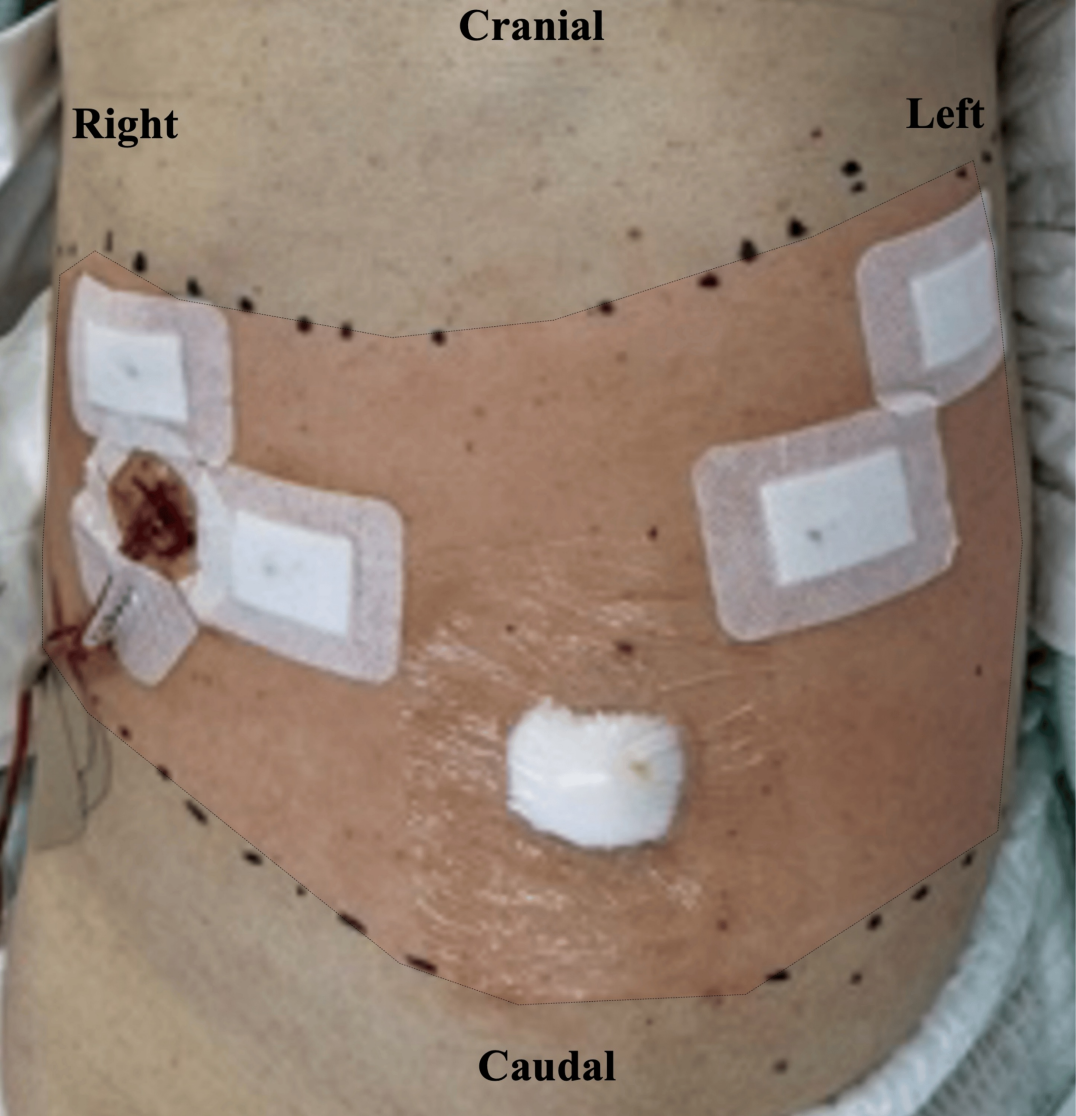
Assessment of sensory blockade pattern after ITPB Results of cold sensation testing 30 minutes after the initial block. The red shaded area with black dots indicates the region of diminished cold sensation (tested with an alcohol swab) from Th8 to Th11 dermatomes bilaterally. Similar sensory blockade patterns were consistently observed until catheter removal on postoperative day 3 ITPB: intertransverse process block

Initial dosing consisted of 0.25% levobupivacaine (10 mL) on each side, which produced adequate sensory blockade from Th8 to Th11, confirmed by cold testing (Figure 4).

Postoperative analgesia was managed without continuous local anesthetic infusion; instead, intermittent boluses of 0.25% levobupivacaine (10 mL on each side) were administered via the catheters twice daily (morning and evening). This was supplemented by intravenous patient-controlled analgesia (IV-PCA) fentanyl (baseline infusion 10 μg/hour, bolus dose 10 μg, lockout time 10 minutes).

The patient reported minimal pain scores (numerical rating scale, NRS, 0-2 at rest and 2-3 with movement) throughout the postoperative period. Notably, the patient did not activate the PCA for additional boluses at any point during recovery. No additional analgesics, such as acetaminophen or NSAIDs, were required throughout the recovery period. The effectiveness of the block was confirmed daily with cold testing, which consistently demonstrated sensory blockade in the Th8-Th11 dermatomes until catheter removal on postoperative day 3 (POD3). After catheter removal, the patient experienced perceptibly increased pain with coughing and during stair climbing at the umbilical port site (NRS < 5 with movement), confirming that the ITPB had provided meaningful analgesia during the initial recovery period. Thanks to effective pain control through POD3, the patient was able to comfortably progress through the early postoperative phase and successfully complete walking rehabilitation without limitations.

The patient achieved early mobilization and was transferred from the ICU to the general ward on POD1 after an uneventful overnight stay. Rehabilitation continued without pain-related limitations throughout the recovery period. No leakage of local anesthetic was observed from the catheter insertion sites. Additionally, there were no adverse effects such as hypotension, motor blockade, urinary retention, or catheter-related complications during the postoperative period. Postoperative laboratory tests showed no deterioration in liver function or coagulation parameters. The patient was discharged home on POD7 without complications.

## Discussion

This case demonstrates the efficacy and safety of ITPB with catheter placement as an alternative to epidural analgesia in patients with suspected portal hypertension. The technique provided excellent pain control, facilitated early mobilization, and avoided the risks associated with epidural procedures in this high-risk patient.

This case adds to the limited literature on bilateral ITPB with catheter placement for intermittent bolus analgesia in patients with alcoholic liver disease and associated vascular abnormalities. Although our patient was classified as Child-Pugh A with a mild model for end-stage liver disease score, the presence of multiple vascular anomalies and a history of variceal bleeding warranted a cautious approach to perioperative pain management.

The use of ITPB rather than epidural analgesia was a critical decision in this case. Patients with portal hypertension develop extensive collateral vessels due to increased portal venous pressure [2]. These collaterals can include engorgement of the epidural venous plexus, increasing the risk of epidural hematoma even with normal coagulation parameters [1]. By placing the needle and catheter in the ITTC rather than the epidural space, we significantly reduced this risk while still providing effective analgesia.

While several similar myofascial plane blocks such as erector spinae plane block (ESPB), midpoint transverse process to pleura block (MTPB), and ITPB function as "paravertebral block by proxy" techniques, our approach differs from the ESPB [6], where the catheter is placed against the transverse process, and local anesthetic is injected into the fascial plane. In our technique, we intentionally advanced the needle tip into the ITTC and placed the catheter within this space. This approach has been validated by anatomical studies showing that the ITTC serves as a conduit to the paravertebral space, allowing for the effective spread of local anesthetic to the spinal nerves [4,5].

Recent literature on "paravertebral by proxy" techniques supports the effectiveness of our approach. Costache et al. [3] introduced this concept, describing blocks that achieve paravertebral spread without direct paravertebral puncture. The MTPB, which targets the same anatomical location (ITTC) as our ITPB, has been shown to provide effective analgesia for thoracic surgery [4]. Our case extends the application of this technique to upper abdominal surgery in a patient with portal hypertension.

It is important to clarify that MTPB and ITPB target the same anatomical structure, the ITTC, but differ primarily in their nomenclature and approach description. MTPB specifically describes targeting the midpoint between the transverse process and pleura, while ITPB refers more broadly to the space between adjacent transverse processes. Both techniques aim to deliver local anesthetic to the paravertebral space via the ITTC, functioning as "paravertebral by proxy" approaches.

Previous studies comparing paravertebral blocks with epidural analgesia have shown comparable efficacy with fewer side effects [7]. Our application of ITPB builds upon these findings, offering the clinical advantage of delivering local anesthetic to the paravertebral space without advancing the needle tip directly into this anatomically sensitive area. This "paravertebral by proxy" approach maintains the analgesic benefits of paravertebral blockade while potentially reducing procedural risks, which is particularly valuable in patients with portal hypertension and vascular abnormalities.

The intermittent bolus technique used in our case was inspired by, though not identical to, the work of Chen et al. [8]. While our approach differed in using manual twice-daily administration rather than hourly programmed boluses, we observed similar benefits in terms of effective analgesia. Our findings suggest that even with a simpler regimen of twice-daily manual boluses, adequate pain control can be achieved without specialized programmable pumps, which may be particularly valuable in resource-limited settings.

While several alternative regional techniques, such as transversus abdominis plane blocks, rectus sheath blocks, or local anesthetic infiltration, might be considered for minimally invasive laparoscopic procedures, these techniques typically provide analgesia limited to the day of surgery when administered as single injections. Continuous wound infiltration was avoided due to infection risk. Moreover, the spread of local anesthetic near surgically manipulated tissues can be unpredictable, potentially resulting in inconsistent analgesia. By contrast, bilateral ITPB with catheter placement offers a mechanism of action similar to epidural analgesia, potentially blocking both somatic and visceral pain through intercostal nerve and sympathetic blockade.

The absence of PCA bolus usage throughout the recovery period highlights the effectiveness of the ITPB in providing adequate analgesia. This is particularly important in patients with liver dysfunction, where minimizing systemic analgesics, including opioids and other pain medications, can prevent hepatic encephalopathy and other complications. While our patient maintained relatively preserved liver function, the benefits of this regional technique would likely be even more pronounced in patients with more severe hepatic impairment. In such cases, the ability to reduce or eliminate the need for acetaminophen, nonsteroidal anti-inflammatory drugs (NSAIDs), and opioids through effective regional anesthesia would provide an even greater clinical advantage by avoiding potential drug-induced liver injury and hepatotoxicity while still ensuring adequate pain control.

An important consideration when performing regional anesthesia in patients with liver disease is the altered pharmacokinetics of local anesthetics. While our patient had relatively preserved liver function, it is worth noting that patients with more advanced cirrhosis (Child-Pugh B or C) may experience significantly reduced clearance of local anesthetics. For instance, research has shown that ropivacaine clearance can decrease by approximately 60% in patients with end-stage liver disease, resulting in elevated unbound drug fractions and prolonged elimination half-lives [9]. In such cases, careful dose adjustment and consideration of adding epinephrine to reduce systemic absorption would be prudent.

In our case, we used levobupivacaine, which, similar to ropivacaine, has a favorable cardiac toxicity profile compared to bupivacaine. With a maximum recommended dose of 2.5-3 mg/kg, our regimen of only 50 mg (0.7 mg/kg) administered twice daily (morning and evening) provided sufficient analgesia while maintaining a considerable safety margin. This suggests that the intermittent bolus technique through ITPB catheters can achieve effective analgesia with significantly lower doses than the maximum recommended limits, representing a safe and efficient approach for patients with liver disease.

The efficacy of the ITPB can be further optimized with careful attention to technical aspects. Nielsen et al. [10] investigated the efficacy of single versus multiple injections in ITPB and found that a single injection approach was noninferior in terms of dermatome coverage. Their findings support our approach of using discrete, targeted boluses rather than continuous infusion, which may help reduce local anesthetic consumption while maintaining analgesic efficacy. The ITPB, in this case, was performed by an anesthesiologist who is a board-certified specialist with the European Society of Regional Anesthesia certification in regional anesthesia techniques. We believe that, with a proper understanding of the relevant anatomy, specifically, recognizing the transverse process acoustic shadow, measuring the distance to the pleura, and identifying the intertransverse ligament-ITPB can be performed more safely than a paravertebral block. However, this technique may not be appropriate for anesthesia trainees without sufficient experience in recognizing these anatomical structures.

The subcutaneous tunneling (5 cm) of the catheters and their secure fixation contributed to the absence of local anesthetic leakage and catheter-related complications. This technical consideration is important for ensuring the sustained effectiveness of catheter-based regional analgesia.

It is important to note that the ITTC is not an open space like the epidural space, which may affect catheter stability and analgesic distribution. In this single case report, we did not obtain radiographic confirmation of the final catheter position after placement, relying instead on functional assessment through cold testing and pain evaluation. Future studies would benefit from X-ray or ultrasound confirmation of catheter positioning to better understand the relationship between catheter location within the ITTC and analgesic efficacy over time.

From a safety perspective, ITPB offers several advantages over neuraxial techniques in patients with liver disease. The technique maintains needle placement in a superficial plane, providing a safer approach than direct paravertebral or epidural puncture. Moreover, ITPB provides clear anatomical landmarks, such as the space between adjacent transverse processes, making the technique more accessible from a technical standpoint. Unlike traditional paravertebral blocks, which often present a steep learning curve for novice practitioners and may not consistently serve as a viable alternative to epidural analgesia, ITPB does not require the advancement of the needle tip into the paravertebral space itself. This anatomical advantage is particularly important in this patient population. Our experience with bilateral ITPB catheter placement demonstrates that this technique offers a safe, technically straightforward approach that provides analgesia comparable to neuraxial blockade without the associated side effects.

Our case adds to the growing body of evidence supporting the use of fascial plane blocks as alternatives to neuraxial techniques in high-risk patients. The successful management of postoperative pain with ITPB in this patient with portal hypertension demonstrates that effective analgesia can be achieved without compromising safety.

## Conclusions

ITPB with catheter placement provided safe and effective postoperative analgesia in a patient with alcoholic liver disease and portal hypertension undergoing laparoscopic partial hepatectomy. This technique enabled adequate pain control, facilitated early mobilization and rehabilitation, and eliminated the need for supplemental opioid analgesia, all while avoiding the risks associated with epidural procedures in this high-risk patient population. Although our patient had relatively preserved liver function (Child-Pugh class A), the presence of significant vascular abnormalities necessitated a cautious approach to perioperative pain management.

As regional anesthesia continues to evolve, ITPB represents a valuable addition to the anesthesiologist's armamentarium, not only for patients with contraindications to neuraxial techniques but also as an option for postoperative pain management in minimally invasive procedures. This approach aligns with modern enhanced recovery protocols by providing effective analgesia while facilitating early mobilization and minimizing systemic medication requirements, making it a promising technique worthy of further investigation through programmed intermittent bolus delivery systems in varying degrees of liver dysfunction.
